# *Solea senegalensis* Bacterial Intestinal Microbiota Is Affected by Low Dietary Inclusion of *Ulva ohnoi*

**DOI:** 10.3389/fmicb.2021.801744

**Published:** 2022-02-08

**Authors:** Isabel M. Cerezo, Milena Fumanal, Silvana T. Tapia-Paniagua, Rocio Bautista, Victoria Anguís, Catalina Fernández-Díaz, Francisco Javier Alarcón, Miguel A. Moriñigo, M. Carmen Balebona

**Affiliations:** ^1^Departamento de Microbiología, Facultad de Ciencias, Ceimar-Universidad de Málaga, Málaga, Spain; ^2^Unidad de Bioinformática – SCBI, Universidad de Málaga, Málaga, Spain; ^3^IFAPA Centro El Toruño, El Puerto de Santa María, Spain; ^4^Departamento de Biología y Geología, Ceimar-Universidad de Almería, Almería, Spain

**Keywords:** microbiome, *Ulva*, algae, *Solea senegalensis*, aquafeed

## Abstract

The inclusion of macroalgae in the diets of farmed fish offers the opportunity for an added-value dietary ingredient to the nutraceutical feed. The composition of algae varies greatly among species. Several *Ulva* species have been considered in aquafeed formulations for different farmed fish, and *Ulva ohnoi* is being applied recently. However, the effects of seaweed dietary inclusion on the host must be evaluated. Considering the important role of the host intestinal microbiota, the potential effects of *U. ohnoi* dietary inclusion need to be studied. In this study, the characterization of the intestinal microbiome of *Solea senegalensis*, a flatfish with high potential for aquaculture in South Europe, receiving *U. ohnoi* (5%)-supplemented diet for 90 days has been carried out. In addition, the functional profiles of bacterial communities have been determined by using PICRUSt, a computational approach to predict the functional composition of a metagenome by using marker gene data and a database of reference genomes. The results show that long-term dietary administration of *U. ohnoi* (5%)-supplemented feed modulates *S. senegalensis* intestinal microbiota, especially in the posterior intestinal section. Increased relative abundance of *Vibrio* jointly with decreased *Stenotrophomonas* genus has been detected in fish receiving *Ulva* diet compared to control-fed fish. The influence of the diet on the intestinal functionality of *S. senegalensis* has been studied for the first time. Changes in bacterial composition were accompanied by differences in predicted microbiota functionality. Increased abundance of predicted genes involved in xenobiotic biodegradation and metabolism were observed in the microbiota when *U. ohnoi* diet was used. On the contrary, predicted percentages of genes associated to penicillin and cephalosporin biosynthesis as well as beta-lactam resistance were reduced after feeding with *Ulva* diet.

## Introduction

Macroalgae have been widely evaluated as a novel ingredient for aquafeed formulation in recent years (Guerreiro et al., 2019; Wang et al., 2019; Yeganeh and Adel, 2019; Naylor et al., 2021). Their nutritional composition include protein, lipids, and high levels of biologically active compounds such as polysaccharides, pigments, polyphenols, and vitamins that might exert beneficial effects on farmed fish (Yaakob et al., 2014; Wells et al., 2017; Moutinho et al., 2018). Firstly, Mustafa and Nakagawa (1995) summarized the role of macroalgae in fish nutrition and then numerous studies have evaluated their potential not only as dietary protein source, but also as functional ingredients in practical diets for a variety of fish species (Yildirim et al., 2009; Güroy et al., 2012; Peixoto et al., 2016, 2017; Zhu et al., 2016). However, the effects detected were dose-dependent and species-specific (Valente et al., 2006).

In this context, *Ulva* species are a good source of protein, minerals, and vitamins, especially vitamin C (Ortiz et al., 2006; García-Casal et al., 2007), and different studies aimed at assessing the dietary inclusion of *Ulva* in a wide range of farmed fish species have been carried out (Wassef et al., 2013; Silva et al., 2015; Valente et al., 2016; Vizcaíno et al., 2016; Magnoni et al., 2017; Kazemi et al., 2018) with positive results on growth performance, innate immune response, feed utilization and overall fish health status. Negative effects on growth have been reported with dietary levels of *Ulva* meal above 10% in aquafeed (Wassef et al., 2005; Diler et al., 2007; Azaza et al., 2008). On the contrary, when added to fish diet at low percentages, benefits such as improved growth, feed efficiency, nutrient utilization, modulation of immune response, and disease resistance have been described in several fish species (Mustafa and Nakagawa, 1995; Wassef et al., 2005; Valente et al., 2006; Ergün et al., 2009; Moutinho et al., 2018; Fumanal et al., 2020).

Although different *Ulva* species have been applied in several areas, the interest on the potential industrial use of *Ulva ohnoi* (Prabhu et al., 2019) and its applicability or its products in aquaculture is recent (Norambuena et al., 2015; Fernández-Díaz et al., 2017; Martínez-Antequera et al., 2021). *U. ohnoi* is widely distributed and easily grown, with fast growth rates even in recirculating fish farming systems (Oca et al., 2019; Kang et al., 2021). Several studies have described effects on the immune system after dietary inclusion of *U. ohnoi* (Fumanal et al., 2020; Kang et al., 2021; Martínez-Antequera et al., 2021). In addition, presence of nutraceutical components such as the polysaccharide ulvan has been identified in *U. ohnoi* extracts (Fernández-Díaz et al., 2017). Thus, this species was considered in the present study based on the possibility to easily obtain enough amounts of algal biomass and its potential use as source of nutraceutical components for Senegalese sole (*Solea senegalensis*).

Senegalese sole is a flatfish with great potential for marine aquaculture due to its high market value and consumer demand. Recent management and technical improvements in *S. senegalensis* culture are leading to important progress in productivity, but there are still unsolved questions regarding nutrition aspects under cultured conditions (Morais et al., 2016), and at present, inclusion of macroalgae in aquafeeds is being considered.

The gastrointestinal (GI) microbiota has a relevant role in animal nutrition, development, modulation of the immune system, and resistance against pathogens (Rawls et al., 2004; Salinas et al., 2006; Semova et al., 2012; Ingerslev et al., 2014a,b; Llewellyn et al., 2014; Wang et al., 2018) and its composition is shaped by different factors such as host species, trophic level, environment, and feeding habits (Romero et al., 2014; Eichmiller et al., 2016; Gonçalves and Gallardo-Escárate, 2017) variations in physiological parameters along the GI (Ye et al., 2014) and functional ingredients or nutraceuticals (Ringø et al., 2016). Composition of *Ulva* spp., as well as *U. ohnoi*, includes polysaccharides such as ulvan (Lahaye and Robic, 2007) and starch (Korzen et al., 2015; Farias et al., 2017). The hydrolysis of those compounds by bacteria has been documented (Michel and Czjzek, 2013; Reisky et al., 2019), so their metabolism by the intestinal microbiota of farmed fish deserve attention. In this context, recently, the administration during a short period of time of a low dietary level of *U. ohnoi* demonstrated to exert a modulation of the intestinal microbiota of farmed Senegalese sole specimens (Tapia-Paniagua et al., 2019). As far as we know, effects of long-term feeding of *U. ohnoi* on the GI microbiota and the potential implications on the functionality are not known. In order to achieve a better understanding of the role of gastrointestinal microbiota in fish health and digestion, further knowledge of the phylogenetic profile and functional capacities of the intestinal microbiota is necessary.

Furthermore, despite the fact that the microbial diversity of Senegalese sole has been described in a recent study (Tapia-Paniagua et al., 2019), there is no solid information about the functional capability of these microbial communities so far. In this sense, some bioinformatic tools can predict microbiome functionality, based on marker genes commonly used for diversity analysis, such as the 16S rRNA. In this work, the predictive software PICRUSt has been used for describing the effects of *U. ohnoi* 5% dietary supplementation on the main functional traits of *S. senegalensis* gut microbiome.

The aim of the present study was to investigate the effects of long-term feeding low dietary inclusion of *U. ohnoi* on the intestinal microbial composition of *S. senegalensis*. In addition, functional profiles of bacterial communities were evaluated by using phylogenetic investigation of communities by reconstruction of unobserved states (PICRUSt) (Langille et al., 2013), and the potential relationship between changes in microbial composition and functionality after *U. ohnoi* feeding was assessed.

## Materials and Methods

### Diet Composition and Preparation

*U. ohnoi* Hiraoka and Shimada, strain UOHN120810 was isolated from the outlet channel of IFAPA El Toruño (El Puerto de Santa María, Cádiz, Spain) fish facilities and vegetative clones maintained in culture. To obtain the *U. ohnoi* biomass needed for the feeding trial (1 kg m^–3^), *U. ohnoi* cultures were up-scaled to 1000-L tanks and grown in modified f/2 medium (Guillard, 1975) with 1.8 mM nitrate and 0.1 mM phosphate prepared with filtered (0.2 μm) natural seawater for 2 weeks under natural photoperiod light. Algae were harvested, rinsed with tap water, freeze-dried, and kept in a dry place until used as ingredient in the experimental diet.

Two isonitrogenous (55% on dry weight basis) and isolipidic (15% on dry weight) experimental diets were manufactured by LifeBioencapsulation SL (Spin-off, Universidad de Almeria, Spain). Ulva diet was formulated to include 5% (w/w) dry *U. ohnoi* biomass. An algae-free diet was used as control. The ingredient composition of experimental diets is shown in Table 1.

**TABLE 1:** Ingredient composition of the experimental diets used in the feeding trial.

*Ingredients* (g kg^–1^ dry weight)	Control diet	Ulva diet
Fishmeal LT^1^	674	660
*Ulva ohnoi* meal		50
Squid meal^2^	50	50
Fish protein hydrolysate^3^	50	50
Krill meal^2^	10	10
Shrimp meal^2^	10	10
Gluten meal^2^	20	20
Soybean protein concentrate^4^	20	20
Fish oil	30	29
Soybean lecithin Maltodextrin	20 46	20 11
Choline chloride^5^	10	10
Vitamin and mineral premix^6^	20	30
Guar gum^2^	15	15
Alginate^2^	15	15

*^1^(69.4% crude protein, 12,3% crude lipid), Norsildemel (Bergen, Norway); ^2^ Local provider (Lifebioencapsulation SL, Almería Spain); ^3^ (81% crude protein, 8.8% crude lipid) Sopropeche (France); ^4^(65% crude protein, 8% crude lipid) DSM (France); ^5^Sigma-Aldrich (Madrid, Spain); ^6^Mineral and vitamin premix according to Vizcaíno et al. (2018). Proximate composition of Control diet: 55.2% crude protein, 12.4% crude lipid, 3.0% fiber, 12.8% ash), and Ulva diet: 55.0% crude protein, 12.0% crude lipid, 3.3% fiber, 13.1% ash.*

Feed ingredients were finely ground and mixed in a vertical helix ribbon mixer (Sammic BM-10, 10-L capacity, Sammic, Azpeitia, Spain) before fish oil and diluted choline chloride being added. All the ingredients were mixed together for 15 min, and, after, water (300 ml kg^–1^) was added to the mixture to obtain homogeneous dough. The dough was passed through a single screw laboratory extruder (Miltenz 51SP, JSConwell Ltd., New Zealand), to form 1–2 mm (diameter) and 2–3 mm (length) pellets. The extruder barrel consisted of four sections and the temperature profile in each section (from inlet to outlet) was 100, 95, 90, and 85°C, respectively. Finally, pellets were dried at room temperature for 24 h and kept in sealed plastic bags at −20°C until use.

### Fish Maintenance

Juvenile Senegalese sole (*S. senegalensis*) (10.7 ± 2.9 g, mean initial body weight) were obtained from a commercial hatchery (Cupimar S.A., San Fernando, Cádiz, Spain) and transported to the research facilities of IFAPA El Toruño (El Puerto de Santa María, Cádiz, Spain). Fish were stocked at 1.5 kg m^–2^ in 6 tanks connected to a closed recirculation consisting of a mechanical filter, a skimmer, ultraviolet light, and a biofilter. Fish were fed daily at 2% fish biomass with an experimental diet considered as control diet for 10 days for acclimatizing the fish to the experimental conditions. After the acclimation period, experimental diets were randomly assigned to triplicate groups. Fish (12.3 ± 2.0 g mean body weight) of each set of three tanks were fed with two different experimental diets: control diet and diet containing *U. ohnoi* 5% (Ulva diet) for 90 days at a rate of 3% of their body weight. Different parameters were monitored during all the experimental period. The temperature, pH, salinity, and oxygen were maintained constant at 19.9 ± 0.7°C; 7.8 ± 0.2; 25.7 ± 1.5‰, and 7.0 ± 0.4 mg L^–1^, respectively. Nitrite and ammonia were checked once a week (values were below 0.1 mg L^–1^).

At the end of the feeding trial, fish were fasted for 12 h before sampling. Soles (*n* = 14) were carefully taken from their respective tanks and transferred to a new tank containing clove oil (200 ppm) to euthanize. Whole intestines of seven fish per treatment were aseptically removed, divided into two equal length sections (anterior and posterior), and stored separately in Trisure, −80°C, until further analysis.

### Sampling Procedures and Sequence Analysis

Individual intestinal samples were collected from *S. senegalensis* specimens with 1 ml of PBS, pH 7.2, and 1 ml aliquot per sample (*n* = 28) was centrifuged (1,000 × *g*, 5 min). Total DNA was extracted from each sample according to manufacturer specifications using Trisure (Bioline, Spain). Afterward, 20 μl of total DNA was precipitated with 2 μl of sodium acetate 3 M and 46 μl isopropanol for purification. Then, DNA was centrifuged for 3 min at 12,000 × *g*, 4°C, supernatants were discarded, and the pellets were rinsed with cold 70% ethanol. After centrifugation for 5–15 min at 12,000 × *g*, 4°C, supernatants were discarded again and pellets were air dried. Finally, DNA was resuspended in water. DNA quality and integrity were visualized by gel electrophoresis. Concentration and purity were determined by using Qubit 2.0 fluorimeter (Thermo Scientific, Germany). Isolated DNA was stored at −20°C until further processing and 30 ng was used for subsequent analyses.

Libraries were constructed by the Ultrasequencing Service of the Bioinnovation Center (University of Malaga, Spain) using the Illumina^®^ MiSeq Platform. Libraries were constructed by using the Illumina MiSeq Platform. Briefly, Illumina paired-end sequencing (2 × 300 bp) of each sample was carried out by using the primers 341F CCTACGGGNGGCWGCAG and 805R GACTACHVGGGTATCTAATCC, targeting V3–V4 regions of 16 S rRNA gene (Klindworth et al., 2013).

Illumina reads were analyzed with FastaQC software (Andrews et al., 2010) to assess sequence quality. Then, reads were processed using a pipeline based on the software package MOTHUR (version 1.39.5) (Schloss et al., 2009). Demultiplexed paired-end reads were merged and processed for primer sequence trimming according to amplicon size (400-600 bp). UCHIME version 4.2. (Effective Tags obtained)^1^ (Edgar et al., 2011) was used to detect and remove chimera; the remaining representative, non-chimeric sequences were aligned and clustered into operational taxonomic units (OTUs) in the Greengenes database (version 13.5) (McDonald et al., 2012) with 97% identity cutoff and the total count threshold was set at 0.005% (Bokulich et al., 2013).

After generating the taxonomic profile of microbiome samples, comparison of taxa present in the samples was carried out. The samples were normalized using the calculation of the rarefaction curves. All statistical analyses were performed using *phyloseq* and *vegan* libraries in R package (McMurdie and Holmes, 2013). To determine the level of sequencing depth, rarefaction curves were obtained by plotting the number of observed OTUs against the number of sequences and Good’s coverage coefficient was calculated. Alpha diversity was estimated based on Chao1, Shannon-Wiener, and Simpson indexes to determine taxonomic and phylogenetic structure diversity, respectively. The results are generally presented at phylum, class, family, and genus taxonomic levels.

Putative microbiota functions were predicted using PICRUSt (version 1.1.3), a tool designed to infer metagenomic information from 16S rRNA amplicon sequencing data using default values (Langille et al., 2013). The metagenomic data resulting from the clustering with the Greengenes database (version 13.5), were entered into the software. The metagenome prediction of the bacterial communities was carried out using the data set calculated after normalizing the number of copies of rRNA 16S to the size of the biome. Nearest Sequenced Taxon Index (NSTI) scores were obtained to assess the precision of predicted metagenomes and ranked at a 97% confidence value using the Kyoto Genome and Gene Encyclopedia Pathway Database (KEGG). All the functional categories were calculated (Kanehisa et al., 2014) and the bacterial functional profiles were compared up to level 3 of the KEGG modules.

### Calculations and Statistical Analysis

Growth parameters and survival rates were calculated according to the following expressions:

Weight gain rate (WGR) (%) = 100 × (final body weight – initial body weight)/initial body weight; Feed conversion ratio (FCR) = Dry feed consumed (g)/wet weight gain (g) and Survival rate (%) = 100 × final fish number/initial fish number.

Normality (Shapiro–Wilk test) and homogeneity of variance (Levene’s test) were tested for all data before differences between the two experimental diets were determined and Student’s *t*-test comparison of means was used. All tests were performed with XLSTAT software and significance was set for *p* < 0.05.

Multivariate analysis of OTU data was performed *via* Principal Coordinate Analysis (PCoA) of OTU profiles using Bray–Curtis metric to depict differences between microbiota of each diet group. In addition, to test the hypothesis of no differences between the microbiota of the GI sections and diets assayed, dissimilarity matrices obtained with the Bray–Curtis index were analyzed by Permutation multivariate analysis of variance (PERMANOVA) with 999 permutations by using PAST software (Hammer et al., 2001) version 3.16.

Linear discriminant analysis (LDA) effect size (LEfSe) (Segata et al., 2011) was used to characterize microbial differences of biological relevance between the diets within the two different GI sections and between sections within the same diet. LEfSe analysis was performed using the Galaxy platform^2^. The taxa whose alpha value was less than 0.05 were selected as significant, first by the Kruskal–Wallis factorial rank-sum test and then by the Wilcoxon paired test, as well as a threshold of |2.0| for the LDA.

STAMP software (Statistical Analysis of Metagenomics Profiles) was used to analyze the differential abundances of predictive functions based on the microbiota of intestinal sections and diets researched, using ANOVA multiple-comparison test with *post hoc* Tukey–Kramer test (*p* corrected < 0.05).

## Results

### Fish Growth and Microbiota Sequencing Overview

Fish mortality during the experimental period was below 5%. Significant decreased final body weight and weight gain rate values were obtained for fish fed with *U. ohnoi* 5%-supplemented diet; however, no differences in FCR were detected based on the diet supplied (see Table 2 for details).

**TABLE 2:** Growth performance of juvenile *Solea senegalensis* specimens.

	Control diet	Ulva diet	*t*	*p*-value
FBW (g)	55.46 ± 1.16a	50.05 ± 1.09b	12.15	0.007
WGR (%)	351.97 ± 9.50a	307.92 ± 8.94b	32.89	0.003
FCR	1.0 ± 0.1	1.1 ± 0.1	–0.63	0.53

*Proximate composition of Control diet: 55.2% crude protein, 12.4% crude lipid, 3.0% fiber, 12.8% ash), and Ulva diet: 55.0% crude protein, 12.0% crude lipid, 3.3% fiber, 13.1% ash. FBW: final body weight; WGR: weight gain rate; FCR: feed conversion ratio. Values are mean ± SD of triplicate tanks. Different letters indicate significant differences between the experimental diets (t-test, t, p < 0.05).*

DNA was extracted from 28 fish intestinal samples, seven *S. senegalensis* specimens per diet and 2 intestinal regions per fish specimen. In total, 5,109,193 raw reads were obtained for both forward and reverse directions after sequencing. Non-specific amplicons not assigned amplicons to the target taxon, and chimeras were removed in the initial quality filtering, and a total of 1,453,999 reads were obtained, with 51,928.53 ± 17,165.22 (mean ± SD) sequences per sample that clustered in a total of 667 OTUs (97% similarity cutoff against the Greengenes database). Sequences were filtered by rarefaction curves to the minimum library size of 30,284 reads (Supplementary Figure 1) and singletons and doubletons were also removed. A total of 190 OTUs were obtained and used for subsequent analysis. Mean Good’s coverage estimator value was 99.94 ± 0.02 (mean ± SD) (ranging from 99.93 to 99.94%), indicating adequate sequencing depth.

Alpha diversity indices were calculated for microbiota data of fish fed both control and Ulva diets in section anterior and posterior. Statistical differences were not observed for microbiota species richness (Chao1) when Ulva diet was administered. Furthermore, no differences in Shannon and Simpson indices were observed between the microbiota of fish specimens fed control or Ulva diet regardless of the GI sections analyzed (see Tables 3A,B for details).

**TABLE 3A:** Alpha diversity of bacterial communities in anterior (A) intestinal tract sections of *Solea senegalensis* specimens.

	Control A	Ulva A	*t*	*p*-value
Chao1	224.50 ± 45.10	152.35 ± 30.75	2.45	0.56
Shannon	1.77 ± 0.26	1.74 ± 0.20	0.22	0.82
Simpson	0.65 ± 0.14	0.69 ± 0.13	–0.46	0.66

*Proximate composition of Control diet: 55.2% crude protein, 12.4% crude lipid, 3.0% fiber, 12.8% ash), and Ulva diet: 55.0% crude protein, 12.0% crude lipid, 3.3% fiber, 13.1% ash. Values represent the mean ± SD. No significant differences were found between Control A vs. Ulva A (t-test, t, p > 0.05).*

**TABLE 3B:** Alpha diversity of bacterial communities in posterior (P) intestinal tract sections of *Solea senegalensis* specimens.

	Control P	Ulva P	*t*	*p*-value
Chao1	142.92 ± 36.18	139.34 ± 59.75	0.11	0.93
Shannon	1.65 ± 0.17	1.84 ± 0.17	–2.45	0.07
Simpson	0.68 ± 0.11	0.74 ± 0.11	–0.99	0.36

*Proximate composition of Control diet: 55.2% crude protein, 12.4% crude lipid, 3.0% fiber, 12.8% ash), and Ulva diet: 55.0% crude protein, 12.0% crude lipid, 3.3% fiber, 13.1% ash. Values represent the mean ± SD. No significant differences were found between Control A vs. Ulva A (t-test, t, p > 0.05).*

### Composition of *Solea senegalensis* Gastrointestinal Microbiota

**FIGURE 1:**
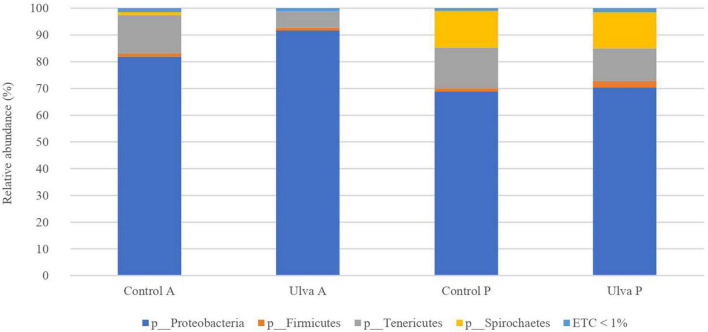
Average of the samples of gut microbiota (relative OTU composition) at phylum level of the gastrointestinal tract of *S. senegalensis* fed control (Control) and *Ulva ohnoi* supplemented (Ulva) diet for 90 days. A, anterior region; P, posterior region. ETC < 1% indicates relative abundance below 1%.

Intestinal microbiota of *S. senegalensis* fed with control and Ulva-supplemented diets was dominated by Proteobacteria, Tenericutes, and Firmicutes in both GI sections (Figure 1). In all the cases, Proteobacteria was the most abundant phylum, ranging from 68.8 to 91.7% relative abundance. In addition, Spirochetes was also present, it being a predominant *phylum* in the posterior GI microbiota of fish fed with both diets (13.5–13.7%).

**FIGURE 2:**
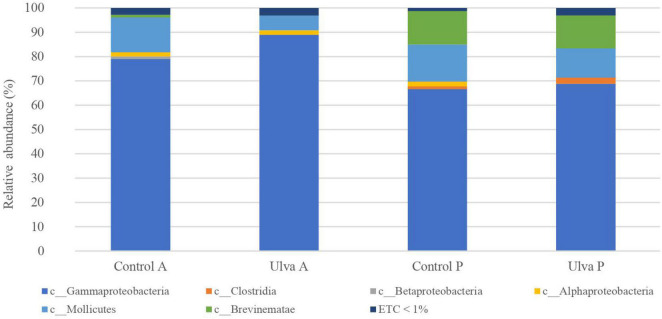
Average of the samples of gut microbiota (relative OTU composition) at class level of the gastrointestinal tract of *S. senegalensis* fed control (Control) and *Ulva ohnoi* supplemented (Ulva) diet for 90 days. A, anterior section; P, posterior section. ETC < 1% indicates relative abundance below 1%.

At the class level (Figure 2 and Supplementary Figure 2), Gammaproteobacteria was the most abundant class in both GI sections (ranging from 66.6 to 88.9%), followed by Mollicutes (from 6.0 to 15.3%). Interestingly, microbiota of *S. senegalensis* specimens fed with Ulva diet showed Mollicutes decreased relative abundance jointly with increased Gammaproteobacteria, it being more evident in anterior sections, although these changes were not reported as statistically significant (Wilcoxon test, *U* = 19, *p* = 0.15) (Figure 2).

**FIGURE 3:**
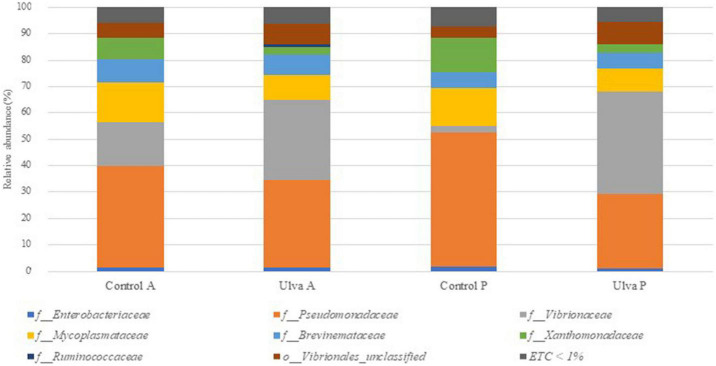
Average of the samples of gut microbiota (relative OTU composition) at family level of the gastrointestinal tract of *S. senegalensis* fed control (Control) and *Ulva ohnoi* supplemented (Ulva) diets for 90 days. A, anterior section; P, posterior section. ETC < 1% indicates relative abundance below 1%.

**FIGURE 4:**
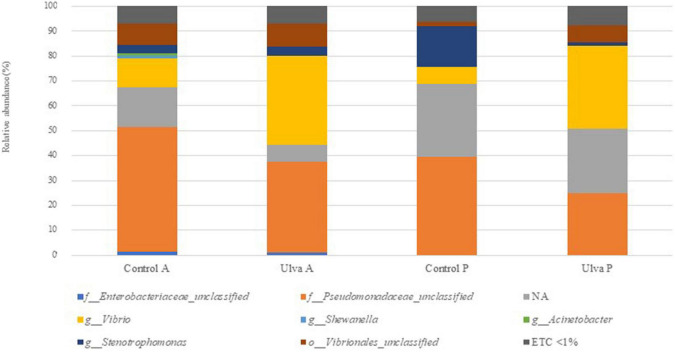
Average of the samples of gut microbiota (relative OTU composition) at genus level of the gastrointestinal tract of *S. senegalensis* fed control (Control) and *Ulva ohnoi* supplemented (Ulva) diet for 90 days. A, anterior section; P, posterior section. NA: OTUs not clustered with the database at the genus level. ETC < 1% indicates relative abundance below 1%.

The taxonomic analysis at the family level showed higher relative abundance of *Pseudomonadaceae* members, it being predominant in both intestinal sections when *S. senegalensis* specimens were fed with control diet (Figure 3 and Supplementary Figure 3). However, abundance of *Vibrionaceae* increased in both intestinal sections of fish fed with Ulva diet compared to those fed with control diet. *Mycoplasmataceae*, *Xanthomonadaceae*, and *Brevinemataceae* were detected in lower abundance percentages in all the samples regardless of the section and diet considered, though relative abundance of the two first was higher in fish receiving control diet. Finally, the study at the genus level (Figure 4 and Supplementary Figure 4) showed *Vibrio* as the most abundant member in the microbiota of specimens fed with Ulva diet, while OTUs identified as “*Pseudomonadaceae_unclassified”* family were the most frequent in the GI microbiota when control diet was supplied, regardless of the section analyzed.

### Modulation of Intestinal Microbiota by *Ulva ohnoi* Diet

**FIGURE 5:**
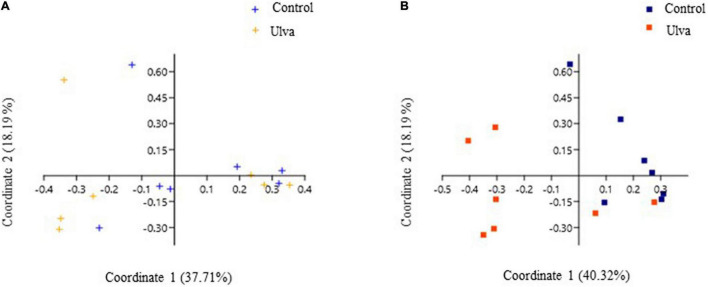
Principal coordinate analysis (PCoA) of bacterial community composition at OTU level based on Bray–Curtis distance matrix. The individual samples are color-coordinated according to the diet received by *S. senegalensis* specimens: control (Control) and *Ulva ohnoi* supplemented (Ulva) diets for 90 days. **(A)** Anterior GI sections, **(B)** Posterior GI sections.

Beta diversity analysis was performed to explore differences in bacterial communities based on the diet received by the fish. Graphical representation of principal coordinate analysis (PCoA) based on Bray–Curtis distances among OTUs detected showed no separate clustering of anterior GI samples associated to the diet (Figure 5A). On the contrary, microbiota samples from posterior GI sections were clearly separated according to the diet received by *S. senegalensis* specimens (Figure 5B). Furthermore, PERMANOVA test confirmed the shift in posterior GI microbiota composition based on the diet, while no significant values were obtained for anterior GI sections used (PERMANOVA, *F* = 3.40, *p* = 0.005, and *p* = 0.49, respectively).

**FIGURE 6:**
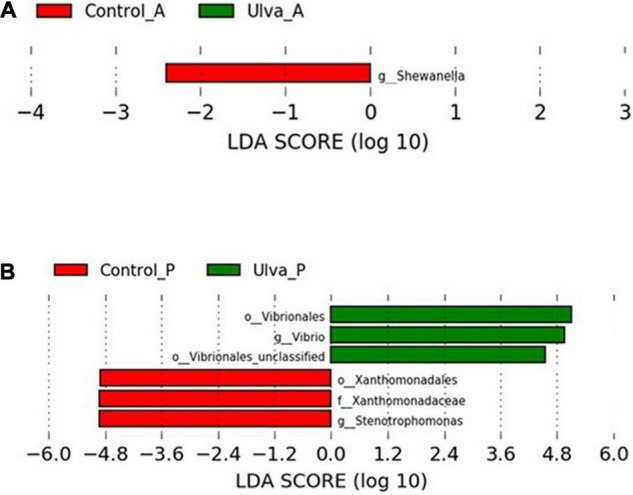
LDA scores for OTUs differentially abundant in the microbiota GI tract of *S. senegalensis* specimens fed control (Control) and *Ulva ohnoi* supplemented (Ulva) diets for 90 days (Kruskal–Wallis and Wilcoxon test, *p* < 0.05 and LDA effect size > | 2.00|). **(A)** anterior section; **(B)** posterior section.

In order to determine the OTUs most likely to explain differences between experimental diets and GI sections, LEfSe analysis (Kruskal–Wallis and Wilcoxon test *p* < 0.05 and LDA effect size > | 2.00|) was carried out. Histograms of the LDA scores computing for differentially abundant OTUs in the GI microbiota of *S. senegalensis* specimens fed with control and Ulva diets and anterior and posterior GI sections are shown in Figure 6.

When comparing the microbiota of anterior sections of fish fed with control and Ulva diets, only *Shewanella* genus were detected as significative (Wilcoxon test, *U* = 7, *p* = 0.02) (Figure 6A). On the contrary, microbiota of the posterior GI sections of fish fed control diet showed significantly increased abundance in *Stenotrophomonas* genus (*Xanthomonadaceae* family, Xanthomonadales order) whereas *Vibrio* genus (previously named taxa, Wilcoxon test, *U* = 0, *p* = 0.001), including Vibrionales order (Wilcoxon test, *U* = 2, *p* = 0.003), was differentially more abundant in the microbiota of fish fed with the algae (Figure 6B).

### Predicted Gastrointestinal Microbiota Function

PICRUSt analysis was performed to predict functional capabilities of intestinal microbial communities detected in *S. senegalensis* specimens receiving the two diets assayed. The low NSTI value (0.068 ± 0.068) obtained in the PICRUSt analysis indicated good accuracy of prediction. A total of 328 KEGG functions corresponding to level 3 KO entries was identified.

**FIGURE 7:**
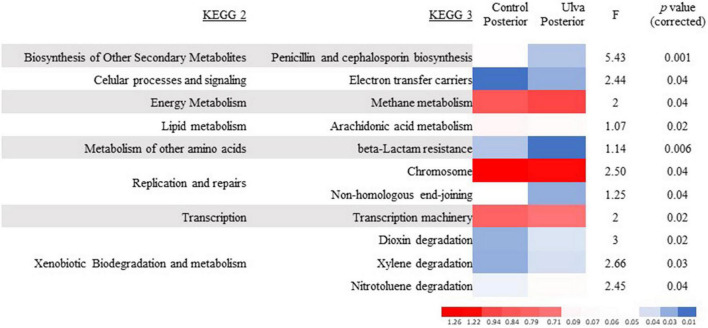
Heatmap showing significant differences (ANOVA, *F*, *p* < 0.05) in microbial functionality (PICRUSt) between the posterior intestinal microbiota of *S. senegalensis* fed control (Control) and *U. ohnoi* supplemented (Ulva) diet for 90 days. Relative abundance of sequences associated with KEGG functions of the samples.

Significant differences in the functional composition of microbial communities of fish fed with both diets were only detected in posterior GI sections. Thus, microbiota of fish fed with *U. ohnoi*-supplemented diet showed increased percentages of genes involved in cellular processes and signaling (electron transfer carriers), energy metabolism (methane metabolism), and xenobiotic biodegradation and metabolism (dioxin, xylene, and nitrotoluene degradation) (see Figure 7 for details). On the contrary, decreased abundance of genes involved in biosynthesis of other secondary metabolites (penicillin and cephalosporin biosynthesis), lipid metabolism (arachidonic acid metabolism), metabolism of other amino acids (beta-lactam resistance), replication and repairs (chromosome and non-homologous end-joining), and transcription (transcription machinery) was predicted for posterior GI microbiota of fish fed with *Ulva* diet compared to those receiving control diet (see Figure 7 for details).

## Discussion

Marine macroalgae represent a promising source of sustainable protein and substances with nutraceutical values, with effects on fish physiology and immune system (Cerezuela et al., 2012; Packer et al., 2016; Fumanal et al., 2020; Ponce et al., 2020; Araújo et al., 2021).

In the present work, decreased growth and feed conversion rates observed in *S. senegalensis* receiving Ulva diet are in agreement with results obtained in previous studies carried out in farmed fish, such as rainbow trout (Yildirim et al., 2009) and *S. senegalensis* (Tapia-Paniagua et al., 2019). The reduced growth observed in fish fed the *Ulva*-supplemented diet has been attributed to the presence of antinutritive factors such as protease inhibitors (Sáez et al., 2013; Vizcaíno et al., 2016, 2019), which could have inhibited digestive proteases in the intestine and adversely affected nutrient uptake.

Microbial ecosystem function and stability are influenced by species and functional group richness (Bell et al., 2005), which, along with the biodiversity, are essential in protecting ecosystem functionality against perturbations (De et al., 2014; Gonçalves and Gallardo-Escárate, 2017). Different studies indicate that diet composition and changes are important factors in the modulation of the GI microbial communities in vertebrates (Askarian et al., 2012; Sullam et al., 2012; Ghanbari et al., 2015; Ringø et al., 2016; Gonçalves and Gallardo-Escárate, 2017). Our results show that the administration of *U. ohnoi* 5%-supplemented diet to *S. senegalensis* did not induce significant differences in the GI microbiota alpha-diversity indices. These results contrast with significantly increased Shannon diversity index values reported when a low *U. ohnoi* inclusion diet was administered to the same fish species for a shorter period (45 days) (Tapia-Paniagua et al., 2019). Shannon index reflects the diversity of the whole microbial community and differences based on the supplemented diet feeding period could be attributed to an adaptation period to the new diet in the microbial communities, it being possible to consider that the microbiota adjustment has been accomplished after 90 days. Thus, similar diversity index may be due to similar number of species and average or evenness of individual distribution among species, though species may be different. In this context, it has been reported that fish age had significant effects on the beta but not alpha diversity of gut microbiota in both freshwater and saltwater habitats (Zhao et al., 2020).

Overall composition detected in *S. senegalensis* GI microbiota in this study corroborate that members of Proteobacteria phylum dominate the microbiota of the GI tract in *S. senegalensis* specimens, whereas Tenericutes represents the second most abundant phylum in this environment (Tapia-Paniagua et al., 2019). Similarly, previous studies at class level have also identified γ-Proteobacteria and Mollicutes in *S. senegalensis* GI tract (Tapia-Paniagua et al., 2014, 2019). The taxonomic analysis at family level demonstrated that *Pseudomonadaceae* and *Vibrionaceae* were the most frequently Proteobacteria detected in the present work, like in the previous study carried out with younger Senegalese sole specimens by Tapia-Paniagua et al. (2019). These results demonstrate the presence of a long-term core microbiota in *S. senegalensis* GI tract.

Diet is considered one of the more relevant factors affecting intestinal microbiota (Webster et al., 2020). Though low-level dietary inclusion of *U. ohnoi* did not influence alpha diversity indices of *S. senegalensis* intestinal microbiota, results obtained indicate that changes in composition of microbial communities occur in posterior intestinal sections after long-term feeding with diet supplemented 5% of this macroalga. Thus, a shift in the presence of *Stenotrophomonas* (*Xanthomonadaceae*) members in favor of *Vibrio* genus was observed in the microbiota of fish fed with the macroalgae-supplemented diet.

*Stenotrophomonas* genus has been reported as a predominant member of the intestinal microbiota of farmed fish (Tapia-Paniagua et al., 2011; He et al., 2016; Puello et al., 2018), including *S. senegalensis* larvae, juvenile specimens (Tapia-Paniagua et al., 2014, 2019), and *S*. *senegalensis* specimens fed with *U. ohnoi* (Tapia-Paniagua et al., 2019). In addition, this genus has been identified as one of the main groups comprising microbiota of marine species such as rorquals (Toro et al., 2021). This genus includes species reported as fish opportunistic pathogens such as *S. maltophilia* (Geng et al., 2010; Abraham et al., 2016), while other strains isolated from microbiota of marine invertebrates (Reina et al., 2019) and fish (Torabi Delshad et al., 2018) have demonstrated the ability to degrade a broad range of N-acyl-homoserine signaling molecules essential for quorum sensing. This signaling system is involved in the control of the expression of genes related to virulence factors, and quenching signal molecules have been reported to result in attenuation of pathogen virulence (Reina et al., 2019). In this context, *Stenotrophomonas* strains have been reported as plant-probiotics showing abilities to degrade and reduce toxic compounds (Mukherjee and Roy, 2016; Zhang et al., 2017) and produce plant growth-promoting factors (Nevita et al., 2018). However, it would be interesting to check similar activities in strains of this genus isolated from fish. Since the libraries of our study were constructed using the V3V4 amplicon reconstruction with a size of 460 bp, the affinity of the taxa makes it very difficult to establish real phylogenetics.

LEfSe analysis showed differentially increased abundance of *Vibrio* in posterior intestinal sections of fish fed with Ulva diet. *Vibrio* genus members have been reported as abundant taxa in the intestinal microbiota of marine fish species (Nayak, 2010; Sullam et al., 2012; Tapia-Paniagua et al., 2019). Although sequences were not assigned to species level in our study, Tapia-Paniagua et al. (2019) observed that sequences related to *Vibrio* genus were mainly assigned to the chitinase-producing *V. scophthalmi*. Some *Vibrio* species are known as pathogenic for fish, but the presence of pathogens as part of the fish microbiome has been previously reported, though its presence did not imply any symptoms of disease (Roeselers et al., 2011; Xing et al., 2013; Wanka et al., 2018). Although it would be very interesting to know if the lineages found in this study are related to pathogenic strains or not, due to the reason explained above about the methodology, it is difficult to establish.

Analysis of the composition of microbial communities does not provide information on the metabolic functionality of the microbiota. PICRUSt analysis has been used to infer functional capabilities of the microbial communities in several studies carried out in fish (Eichmiller et al., 2016; Cornejo-Granados et al., 2017; Zhang et al., 2017; Huang et al., 2018; Kim et al., 2021). It has afforded to infer functional differences according to different intestinal sections (Zhang et al., 2017) and functional changes associated to the trophic level (Liu et al., 2016). As far as we know, this is the first work addressing the study of the functional potential of bacterial communities in the intestinal tract of *S. senegalensis*.

Predicted functions of microbiota did not show significant changes in anterior intestinal sections of fish fed control and *U. ohnoi* diets. However, along with differences in microbial composition, differences were predicted in microbiota functionality in the posterior intestinal tract. In these sections, predicted functionality of microbiota of fish receiving Ulva diet showed increased abundance of genes involved in xenobiotic biodegradation and metabolism (KEGG 2 level). Due to the limitation of the fishmeal to make aquafeeds, the dietary inclusion of higher levels of plant material is increasing. This substitution in the feed also introduces unwanted substances, some of them as xenobiotics (Sanden et al., 2018; Olsvik et al., 2019; Oliveira and Vasconcelos, 2020).

Gut microbiome has an important role not only on dietary nutrient digestion, but also on xenobiotics metabolism. Thus, activity of intestinal microbiota may result in the inactivation of xenobiotics or, on the contrary, lead to bioactivation of some compounds resulting in increased toxicity (Collins and Patterson, 2020). On the other hand, it has been reported that exposure to dioxin-type polychlorinated biphenyls (PCB) results in disrupted gut microbiota and host metabolism as well as intestinal inflammation (Petriello et al., 2018; Sun et al., 2019). The influence of xenobiotics on fish microbiota composition has already been documented. In fathead minnow (*Pimephales promelas*), the exposure to benzo[a]pyrene results in shifts in microbial composition and enrichment in hydrocarbon-degrading taxa (DeBofsky et al., 2020).

In the present study, the enrichment in microbial communities capable to degrade xenobiotics in *S. senegalensis* fed with *U. ohnoi* diet may be considered a positive trait. In this way, improved intestinal mucosa has been reported in *S. senegalensis* fed with this macroalgae (Vizcaíno et al., 2019). Similarly, Tarnecki et al. (2019) suggested that greater xenobiotic degradation ability of microbiota may contribute to increased Common snook (*Centropomus undecimalis*) survival in larvae treated with probiotics.

On the other hand, decreased abundance of genes related to beta-lactam resistance (KEGG 3 level) was predicted in the microbiota of fish fed with *Ulva*-supplemented diet compared to the control diet group. In a recent study, Kokou et al. (2020) have reported the existence of a core microbiota in European seabass (*Dicentrarchus labrax*) intestinal tract after treatment with antibiotics, though they did not elucidate if these populations contained increased numbers of antibiotic resistance genes. Increasing microbial resistance to antibiotics represents a global concern due to their potential transmission not only to animal but also to human pathogens. In addition, discharge of aquaculture waste containing antibiotic-resistant bacteria results in environmental impact, highlighting the importance of the presence of antibiotic resistance genes in the fish gut microbiome (Kokou et al., 2020). Lower abundance of antibiotic resistance genes predicted in the microbiota of *S. senegalensis* receiving dietary *U. ohnoi* could contribute to reduced antimicrobial resistance dispersion in aquaculture environments.

This finding was accompanied with higher abundance of genes related to penicillin and cephalosporin biosynthesis in fish fed with diet devoid of *U. ohnoi*. It may be considered that lower levels of antibiotics can be expected to be jointly found with lower resistance levels thanks to a selection pressure process.

On the other hand, presence of antibiotics in an ecosystem influences the populations comprising microbial communities. Whether differences observed in the microbiota composition of fish fed with control and Ulva diet may also reflect the presence of genes involved in antibiotic biosynthesis needs to be elucidated.

In summary, long-term dietary inclusion of *U. ohnoi* results in modulation of *S. senegalensis* intestinal microbiota, especially in posterior intestinal sections. In addition, influence of the diet on the intestinal functionality of this fish species has been studied for the first time. Increased abundance of predicted genes involved in xenobiotic biodegradation and metabolism has been observed in the microbiota when *U. ohnoi* diet was used. On the contrary, predicted percentages of genes associated to penicillin and cephalosporin biosynthesis as well as beta-lactam resistance were reduced after feeding Ulva diet. Results reported represent the starting point in the study of the effects of diets on potential changes in *S. senegalensis* intestinal tract microbial community functionality.

## Data Availability Statement

The datasets presented in this study can be found in online repositories. The names of the repository/repositories and accession number(s) can be found below: https://www.ncbi.nlm.nih.gov/, PRJNA765168.

## Ethics Statement

The animal study was reviewed and approved by European Union (2010/63/UE) and the Spanish legislation (RD 1201/2005 and RD 53/2013) for the use of laboratory animals. All procedures were authorized by the Bioethics and Animal Welfare Committee of the Institute of Agricultural and Fisheries Research and Training (IFAPA) and given the registration number 17/11/2016/171 according to the national authorities for regulation of animal care and experimentation.

## Author Contributions

IC and RB performed the bioinformatic analysis and presented the results. ST-P and MF carried out the sampling and DNA extraction. CF-D and VA participated in sampling, cultivation of the algae, and fish maintenance. FA designed and prepared the aquafeeds. MM and MB performed the interpretation of results and wrote the manuscript. MB, MM, CF-D, and FA designed the work. All authors contributed to the discussion and final writing.

## Conflict of Interest

The authors declare that the research was conducted in the absence of any commercial or financial relationships that could be construed as a potential conflict of interest.

## Publisher’s Note

All claims expressed in this article are solely those of the authors and do not necessarily represent those of their affiliated organizations, or those of the publisher, the editors and the reviewers. Any product that may be evaluated in this article, or claim that may be made by its manufacturer, is not guaranteed or endorsed by the publisher.

## Footnotes

1
https://drive5.com/uchime

2
http://huttenhower.sph.harvard.edu/galaxy/
