# Supplementary figures and images for: Solea senegalensis Bacterial Intestinal Microbiota Is Affected by Low Dietary Inclusion of Ulva ohnoi
Source: Front Microbiol. 2022 Feb 8;12:801744. doi: 10.3389/fmicb.2021.801744 (PMC8861459; doi:10.3389/fmicb.2021.801744)

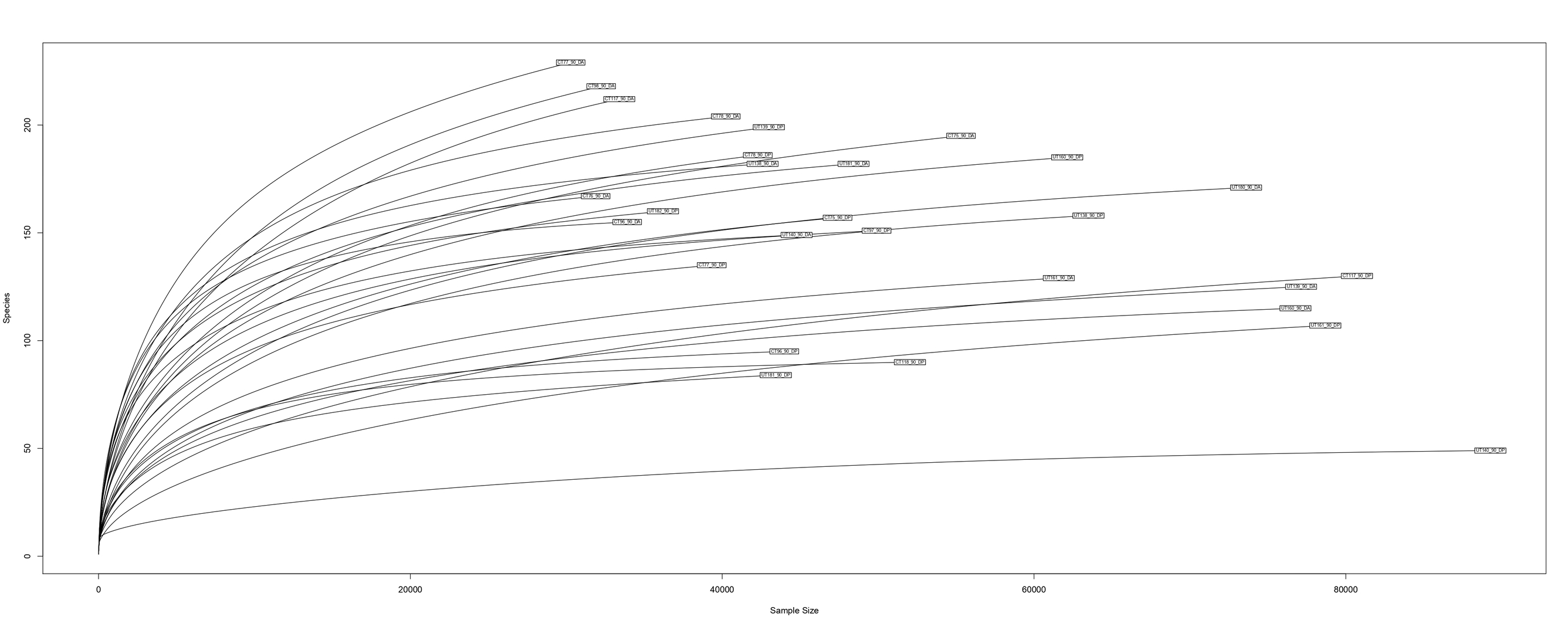

Supplement: Supplementary file 1 [file Image_1.JPEG]

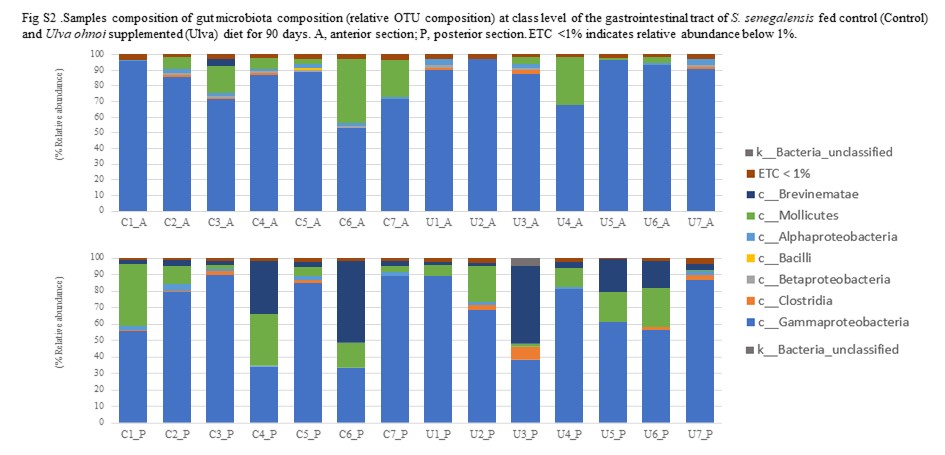

Supplement: Supplementary file 2 [file Image_2.jpg]

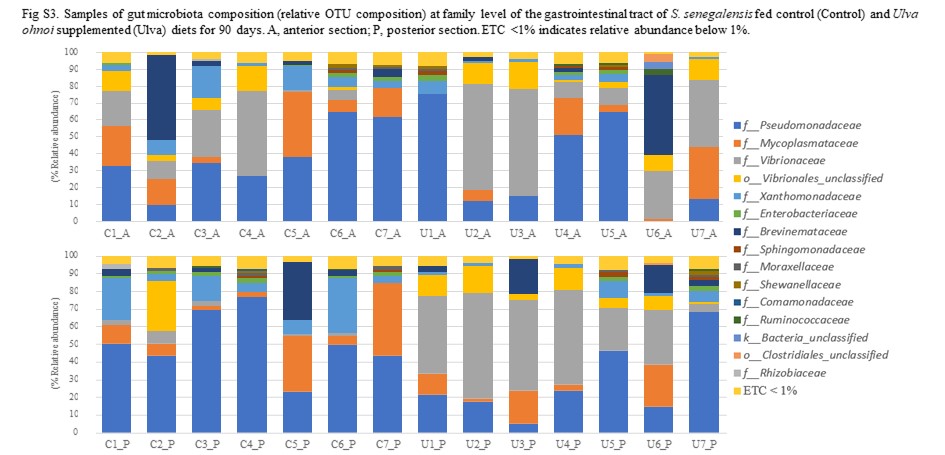

Supplement: Supplementary file 3 [file Image_3.jpg]

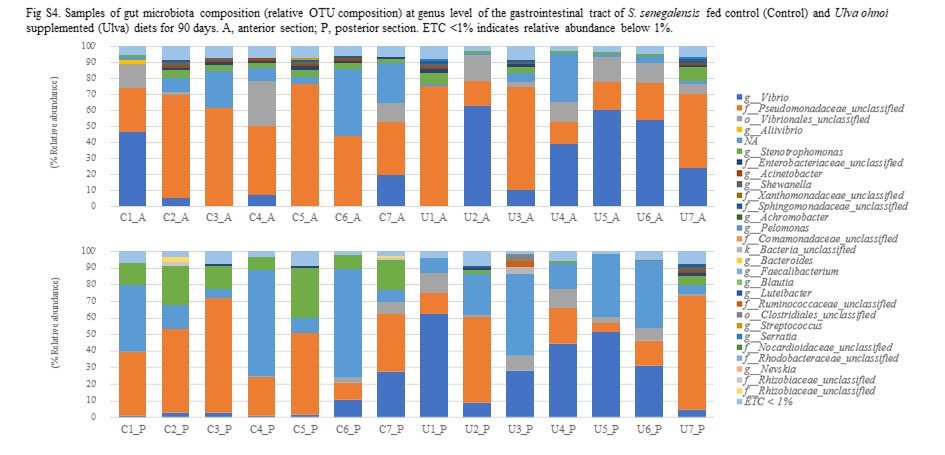

Supplement: Supplementary file 4 [file Image_4.jpg]
